# Pro-Oxidant Enzymes, Redox Balance and Oxidative Damage to Proteins, Lipids and DNA in Colorectal Cancer Tissue. Is Oxidative Stress Dependent on Tumour Budding and Inflammatory Infiltration?

**DOI:** 10.3390/cancers12061636

**Published:** 2020-06-20

**Authors:** Justyna Zińczuk, Mateusz Maciejczyk, Konrad Zaręba, Anna Pryczynicz, Violetta Dymicka-Piekarska, Joanna Kamińska, Olga Koper-Lenkiewicz, Joanna Matowicka-Karna, Bogusław Kędra, Anna Zalewska, Katarzyna Guzińska-Ustymowicz

**Affiliations:** 1 Department of Clinical Laboratory Diagnostics, Medical University of Bialystok, Waszyngtona 15a, 15-269 Białystok, Poland; violetta.dymicka-piekarska@umb.edu.pl (V.D.-P.); joanna.kaminska@umb.edu.pl (J.K.); o.koper@wp.pl (O.K.-L.); matowic@umb.edu.pl (J.M.-K.); 2 Department of Hygiene, Epidemiology and Ergonomics, Medical University of Bialystok, Mickiewicza 2c, 15-222 Białystok, Poland; 3 2nd Clinical Department of General and Gastroenterological Surgery, Medical University of Bialystok, M. Skłodowskiej-Curie 24a, 15-276 Białystok, Poland; konrad.zareba@umb.edu.pl (K.Z.); chgastro@umb.edu.pl (B.K.); 4 Department of General Pathomorphology, Medical University of Bialystok, Waszyngtona 13, 15-269 Białystok, Poland; anna.pryczynicz@umb.edu.pl (A.P.); katarzyna.guzinska-ustymowicz@umb.edu.pl (K.G.-U.); 5 Independent Laboratory of Experimental Dentistry, Medical University of Bialystok, M. Skłodowskiej-Curie 24A, 15-276 Białystok, Poland; anna.zalewska1@umb.edu.pl

**Keywords:** colorectal cancer, redox biomarkers, oxidative stress, antioxidants

## Abstract

This study is the first to assess redox homeostasis in patients with colorectal cancer (CRC) in respect to histopathological parameters associated with the tumour microenvironment such as tumour budding and inflammatory infiltration. Pro-oxidant enzymes (NADPH oxidase (NOX), xanthine oxidase (XO)), antioxidant barrier (Cu,Zn-superoxide dismutase (SOD), catalase (CAT), glutathione peroxidase (GPx), glutathione reductase (GR), reduced glutathione (GSH)), redox status (total antioxidant (TAC)/oxidant status (TOS)) and oxidative damage products (advanced glycation end products (AGE), advanced oxidation protein products (AOPP), malondialdehyde (MDA) and 8-hydroxydeoxyguanosine (8-OHdG)) were determined in both the normal and cancerous tissue of 29 CRC patients. The activity of NOX (*p* < 0.01) and XO (*p* = 0.01), as well as SOD (*p* < 0.0001), CAT (*p* < 0.0001) and TAC level (*p* < 0.01) were significantly higher in tumour tissue than in normal colon mucosa. Oxidative damage products (AGE—*p* < 0.01, AOPP—*p* < 0.001, MDA—*p* < 0.001, 8-OHdG—*p* < 0.0001) were also higher in cancerous colon tissue. Furthermore, we observed that CAT (*p* < 0.05) and XO (*p* < 0.05) activity depends on the intensity of inflammatory infiltration. Oxidative stress index (OSI) (*p* < 0.05) and MDA (*p* < 0.01) values were significantly higher in patients with tumour budding (TB) > 5 versus cases with TB < 5. However, OSI level did not differ significantly between cancer and normal tissue. Our results confirm that CRC is associated with enzymatic/non-enzymatic redox imbalance and increased oxidative damage to proteins, lipids and DNA. The determination of these biomarkers could be useful for the evaluation of the tumour progression.

## 1. Introduction

Colorectal cancer (CRC) is one of the cancers with the highest dynamics of incidence in the world. According to GLOBOCAN database from 2018, colorectal cancer was classified as third most common type of cancer in the world among men and second most frequent one in women. Moreover, CRC is responsible for 9.2% of all cancer deaths, which places it second in terms of mortality among all cancers [1]. Despite screening tests, such as colonoscopy and faecal occult blood tests, about 20–25% of patients have remote metastases when diagnosed with CRC. Around 85% of liver metastases are diagnosed within one year, 94% within two years, and 97.5% within the first three years after being diagnosed with primary CRC [2]. In order to improve such unfavourable statistics, it is crucial to understand the mechanisms involved in CRC carcinogenesis. Indeed, it may facilitate the early detection of cancer, prevent its progression into an advanced stage, and enable the application of treatment which would significantly reduce CRC mortality.

In the last two decades, scientists studying carcinogenesis have been focusing on reactive oxygen species (ROS) and redox balance. Oxidative stress affects the development of cancer by leading to insensitivity of cancer cells to antiproliferative signals and apoptosis, anchorage-independent cell growth, and modification of cancer cell invasion and migration through epigenetic and metabolic mechanisms. ROS also participate in tumour angiogenesis through the release of vascular endothelial growth factor (VEGF) and angiopoietin [3]. High levels of ROS in cancer cells may result from enhanced basal metabolic activity, peroxisome activity, increased oncogene expression, mitochondrial dysfunction (due to hypoxia or mitophagy), cytokine signalling as well as increased activity of NADPH oxidase (NOX), cyclooxygenases or lipoxygenases which are considered as ROS sources [3]. It has been discovered that ROS may modulate the tumour microenvironment responsible for increasing aggressiveness and metastatic dissemination.

In our previous work, we showed that serum catalase (CAT) and plasma malondialdehyde (MDA) may be potential non-invasive biomarkers indicating tumour invasion depth or the presence of lymph node metastasis [4]. However, the precise underlying mechanism of oxidative stress in cancer cells and molecular pathogenesis of CRC remains unknown. Thus, the aim of our study was to evaluate the redox status, enzymatic and non-enzymatic antioxidants as well as oxidative damage to proteins, lipids and DNA in tumour tissue compared to normal adjacent mucosa in colorectal cancer patients. We are also the first to assess the redox parameters in CRC patients in respect to histopathological parameters associated with the tumour microenvironment, such as tumour budding and inflammatory infiltration.

## 2. Materials and Methods

This research was approved by the Bioethics Committee of the Medical University of Bialystok, Poland (permission number R-I-002/48/2019). After a thorough explanation of the purpose of the study and possible risks, all the qualified patients consented in writing to participate in the experiment. The study was conducted in accordance with the World Medical Association Declaration of Helsinki for ethical principles for medical research involving human subjects.

### 2.1. Patients and Tissue Samples

The study was performed on a group of 29 patients treated surgically for colorectal cancer in the 2nd Clinical Department of General and Gastroenterological Surgery at the Medical University of Bialystok Clinical Hospital in the years 2017–2019. The patients were selected based on the following criteria: patients of both genders without comorbidity who had not been treated with radio- or chemotherapy before the surgery. The exclusion criteria in patients with CRC included any systemic or autoimmune diseases (diabetes, insulin resistance, hypertension, coronary heart disease, rheumatoid arthritis and psoriasis), as well as lung, thyroid, liver, kidney, gastrointestinal and infectious diseases (HCV and HIV infection) and immunity disorders. Additionally, smokers and patients who had taken drugs (antibiotics, non-steroidal anti-inflammatory drugs, glucocorticosteroids, vitamins and dietary supplements) within the preceding 3 months were excluded from the study.

The time from diagnosing a patient with cancer to their surgery varied from a minimum of two days to a maximum of four weeks. The study material was collected from all patients during the surgical resection of the tumour. Each patient had the following samples taken: normal mucosa (adjacent non-tumour tissue) and tumour tissue. Normal mucosa was collected from the corresponding adjacent tissue away from the tumour border (histologically examined). After resection, the obtained material was placed in a container with liquid nitrogen and frozen at −80 °C. Part of the tissue was no frozen and used immediately to determine the activity of pro-oxidant enzymes.

### 2.2. Histopathological Analysis

**Figure 1:**
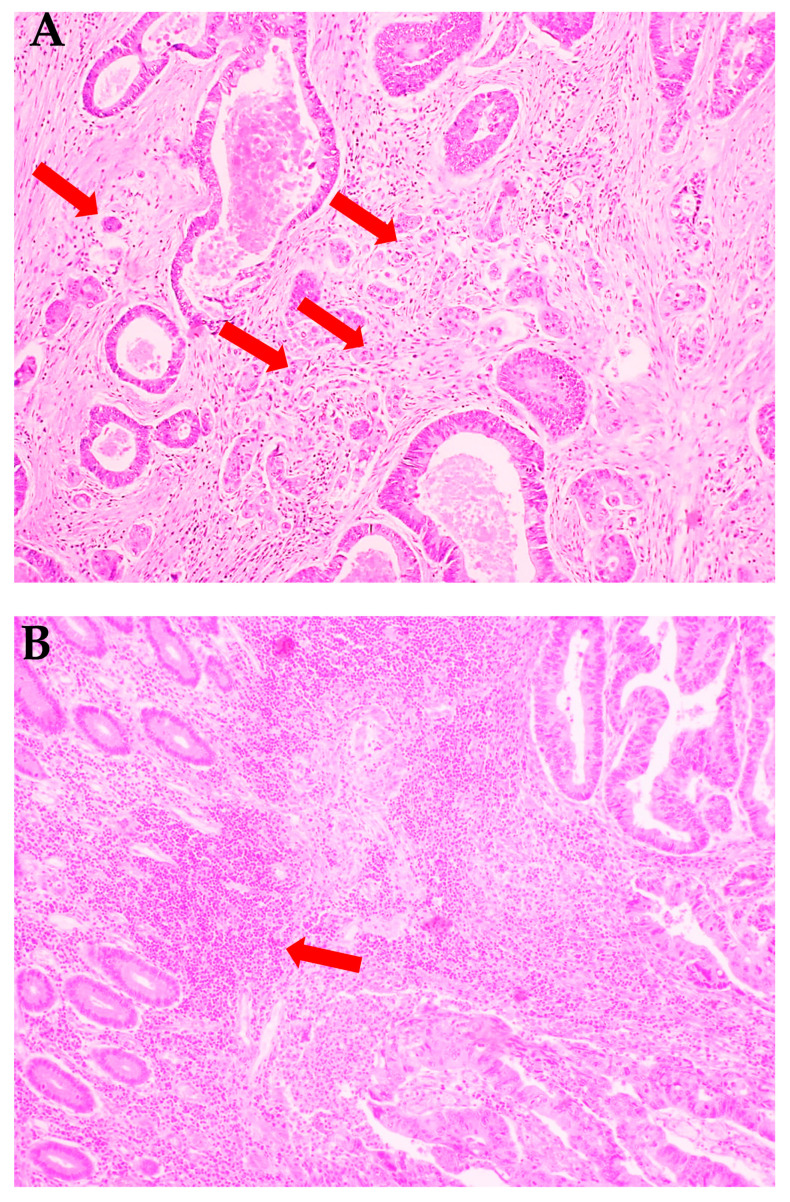
Histological images of colorectal cancer. (**A**) Colorectal adenocarcinoma with budding foci (red arrows). (**B**) Colorectal adenocarcinoma with strong inflammatory cells infiltration (red arrow). H+E staining. 200× magnification.

Histopathological diagnosis was established based on haematoxylin and eosin (H+E) staining and included the following parameters: histological type and the grade of histological malignancy according to the World Health Organization guidelines [5], tumour stage according to the TNM classification standard of the Union for International Cancer Control [6], tumour inflammatory infiltration according to Jass’s classification [7] as well as tumour budding (TB), poorly differentiated clusters (PDC), and areas of poorly differentiated components (APDC) according to Ueno’s classification [8]. Tumour budding has been defined as the presence of individual cells or small clusters (up to 5) of tumour cells at the invasive front of cancer (Figure 1A). Tumours without budding foci were described as 0, with <5, 5 to 9, and >10 budding foci and classified as 1, 2, and 3, respectively. Tumours defined as 0 and 1 were described as low-grade budding, whereas those defined as 2 and 3 were described as high-grade budding. Poorly differentiated clusters (PDC) were defined as cancer clusters in the stroma composed of 5 cancer cells and lacking a gland-like structure. Tumours without clusters were described as 0, with <5, 5 to 9, and >10 clusters were classified as 1, 2, and 3, respectively. Areas of poorly differentiated components were reported as 0—without clusters, while those with <10 clusters and those with >10 clusters were classified as 1 and 2, respectively [8].

The characteristics and details of the study group are presented in Table 1.

Each tumour was cut along a line parallel to the longest tumour axis. Thus, 4 to 8 slices containing cancer cells and the adjacent macroscopically unchanged tissues of 1–1.5 cm in size were collected. The tissues were fixed in 10% buffered formalin for no longer than 24 h. The specimens were embedded in paraffin at a temperature of 56 °C. Paraffin blocks were cut into 4-μm-thick sections with a microtome Microm H340. The obtained sections were stained with haematoxylin and eosin, and reviewed by two independent pathologists under a microscope Olympus CX22 with 200× and 400× magnification.

### 2.3. Preparation of the Tissues 

On the day of biochemical assays, the tissue samples were slowly thawed at 4 °C, fragmented, weighed, and divided into two equal parts each. One of the parts was diluted in ice-cold phosphate-buffered saline (PBS, 0.02 M, pH 7.4) at a ratio of 1:10 (w/v). Tumour and non-tumour tissues were homogenized on ice with a glass tissue homogenizer (Omni TH, Omni International, Kennesaw, GA, USA), sonicated twice (1800 J/sample, 20 s × 3; UP 400S, Hielscher, Teltow, Germany), and then centrifuged (12,000× *g*, 20 min, 4 °C; MPW Med Instruments, Warsaw, Poland) to collect the supernatant and to be immediately assayed [9]. In order to prevent sample oxidation and proteolysis, butylated hydroxytoluene (BHT; 10 μL 0.5 M BHT in acetonitrile/1 mL PBS) and proteolysis inhibitors (Complete Mini Roche, Mannheim, Germany) were added [10].

### 2.4. Determination of Redox Markers

All the assays were performed in duplicate samples. The absorbance/florescence was measured using Infinite M200 PRO Multimode Microplate Reader (Tecan, Männedorf, Switzerland). The results were standardized to 100 mg of total protein. The content of total protein was estimated colorimetrically at 562 nm wavelength via the bicinchoninic acid (BCA) method [11]. A commercial kit was used according to the manufacturer’s instructions (Thermo Scientific PIERCE BCA Protein Assay, Rockford, IL, USA).

#### 2.4.1. Pro-Oxidant Enzymes

The activities of pro-oxidant enzymes, NADPH oxidase (NOX) and xanthine oxidase (XO) were evaluated immediately after sample collection. The freshly harvested tissues were homogenized and centrifuged and the supernatant obtained was used for determinations. NOX (EC 1.6.3.1) activity was analysed by the luminescence method using lucigenin as an electron acceptor [12]. One unit of NOX activity was defined as the amount of the enzyme required to release 1 nmol of superoxide anion per 1 min. XO (EC 1.17.3.2) activity was estimated using the colorimetric method by measuring the increase in uric acid absorbance at 290 nm wavelength [13]. One unit of XO activity was defined as the amount of the enzyme required to release 1 μmol of uric acid per 1 min.

#### 2.4.2. Antioxidant Enzymes and Non-Enzymatic Antioxidants

To study the antioxidant barrier, antioxidant enzymes (catalase (CAT), glutathione peroxidase (GPx), glutathione reductase (GR), superoxide dismutase-1 (SOD-1)) as well as non-enzymatic antioxidants (reduced glutathione (GSH)) were evaluated.

CAT (EC 1.11.1.6) activity was analysed using the colorimetric method by measuring the rate of hydrogen peroxide (H_2_O_2_) decomposition at 240 nm wavelength [14]. One unit of CAT activity was defined as the amount of the enzyme catalysing the decomposition of 1 mmol H_2_O_2_ per 1 min. GPx (EC 1.11.1.9) activity was estimated using the colorimetric method by measuring the oxidation of NADPH (reduced form of nicotinamide adenine dinucleotide phosphate) at 340 nm [15]. One unit of GPx activity was defined as the amount of the enzyme catalysing the oxidation of 1 mmol NADPH per 1 min. GR (EC 1.8.1.7) activity was determined using the colorimetric method by measuring the decrease in NADPH absorbance at 340 nm [16]. One unit of GR activity was defined as the quantity of the enzyme catalysing the oxidation of 1 μmol NADPH per 1 min. SOD-1 (E.C. 1.15.1.1) activity was evaluated colorimetrically by measuring the inhibition of adrenaline oxidation at 480 nm wavelength [17]. One unit of SOD activity was defined as the amount of the enzyme inhibiting adrenaline oxidation by 50%.

Glutathione concentration was determined colorimetrically using the enzymatic reaction with 5,5-dithiobis-(2-nitrobenzoic acid) (DTNB), NADPH, and GR [18]. The reaction results in the formation of a product that absorbs at 412 nm. The reduced glutathione (GSH) concentration was calculated from the difference between the concentration of total glutathione and oxidized glutathione (GSSG). Oxidation/reduction potential (redox state) was calculated based on the formula = [GSH]^2^/[GSSG] [19]. 

#### 2.4.3. Redox Status

To assess the redox status, total antioxidant capacity (TAC), total oxidant status (TOS), and oxidative stress index (OSI) were evaluated.

TAC level was determined using the colorimetric method by measuring changes in ABTS^+^ (2,2′-azino-bis-3-ethylbenzothiazoline-6-sulfonate) absorbance at 660 nm [20]. TOS level was analysed using the colorimetric method by measuring the oxidation of ferrous ion to ferric ion in the presence of oxidants in a sample [21]. OSI was calculated using the formula: OSI = [TOS]/[TAC] × 100% [22].

#### 2.4.4. Oxidative Damage to Proteins, Lipids and Nucleic Acids

Oxidative damage to proteins (advanced glycation end products (AGE), advanced oxidation protein products (AOPP)), lipids (malondialdehyde (MDA)] and nucleic acids [8-hydroxydeoxyguanosine (8-OHdG)) was evaluated.

AGE content was estimated using the fluorimetric method by measuring AGE-specific fluorescence at 350/440 nm wavelength [23]. Immediately prior to the assay, samples were diluted in 0.02 M PBS, pH 7.4 (1:5, v/v). AOPP concentration was determined colorimetrically by measuring iodide ion oxidizing capacity of the sample at 340 nm [23]. Immediately before the assay, samples were diluted in 0.02 M PBS, pH 7.4 (1:5, v/v). MDA concentration was analysed colorimetrically at 535 nm using the thiobarbituric acid reactive substances (TBARS) method and 1,3,3,3-tetraethoxypropane as a standard [24]. The 8-OHdG level was determined colorimetrically with commercial ELISA kits (Cayman Chemical, Miami, FL, USA; USCN Life Science, Wuhan, China, respectively), according to the manufacturer’s instructions.

### 2.5. Statistical Analysis

Statistical analysis was performed using the GraphPad Prism (GraphPad Software, La Jolla, CA, USA). The Shapiro–Wilk test was used to examine the distribution of results. For normal distribution, the Student’s *t*-test was used. In the case of the lack of normal distribution, the Mann–Whitney U test was used. The data are presented as median (minimum–maximum). The correlations between the measured parameters were analysed using Spearman’s correlation coefficient. Statistical significance was established at *p* < 0.05.

The results show only comparisons of tumour vs. normal cancer tissue and other statistically significant differences. All comparisons are presented in the Tables S1 and S2. 

## 3. Results

### 3.1. Clinical Characteristics

The study included 29 patients with colorectal cancer. The tumour differentiation grade in all colorectal cancer patients was G2 (moderately differentiated). Most of the subjects (25 people) had adenocarcinoma, and four patients had mucinous adenocarcinoma. Over 65% patients had a pT2 stage of tumour which had grown through the muscularis propria and into the subserosa as well as into tissues surrounding the colon. The percentage of patients without lymph node (N0) and distant metastasis (M0) was 32.1% and 89.7%, respectively. Meanwhile, 58.6% patients had a venous invasion, which is defined as “tumour present within an endothelial-lined space either surrounded by a rim of muscle or containing red blood cells” [25]. The majority of our study group consisted of patients without neural invasion (72.4%). Neural invasion is defined as presence of tumour cells within the perineurial space or in direct contact with nerve fascicles [26].

Detailed characteristics of the study groups are summarised in Table 1.

### 3.2. Pro-Oxidant Enzymes

**Figure 2:**
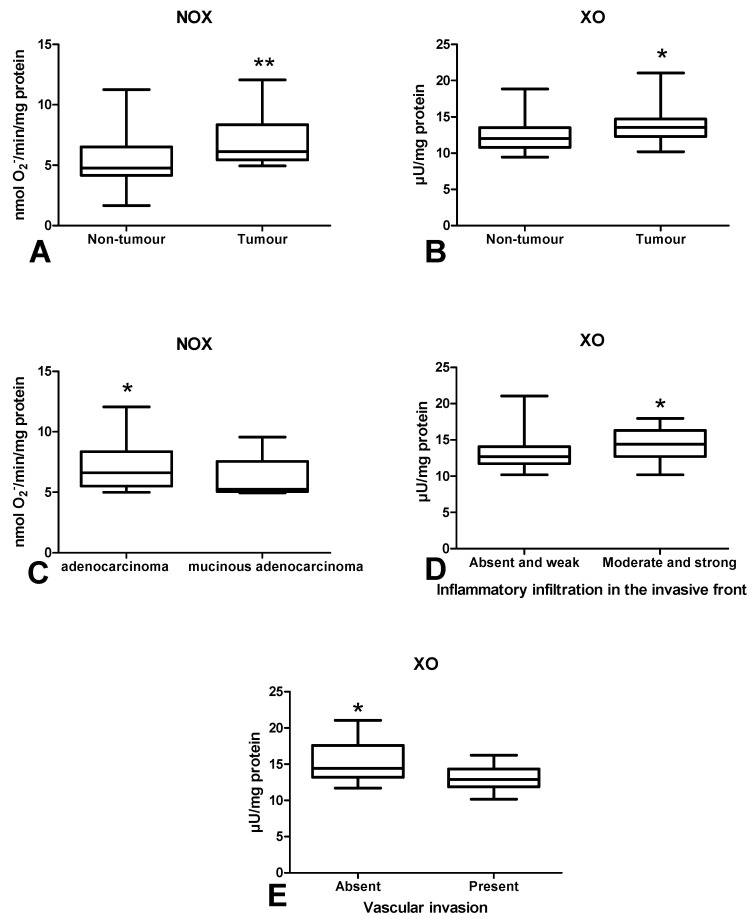
Comparison of: NOX (**A**) and XO (**B**) activity between colorectal cancer tissue (*n* = 29) and normal mucosa (*n* = 29); NOX activity (**C**) between adenocarcinoma and mucinous adenocarcinoma; XO activity (**D**) depending on the intensity of inflammatory infiltration in the invasive front of the tumour; XO activity (**E**) between colorectal tissue with and without vascular invasion. The data are presented as median (minimum–maximum). Abbreviations: NOX—NADPH oxidase, XO—xanthine oxidase. * *p* < 0.05, ** *p* < 0.01.

Determination of NADPH oxidase (NOX) activity in colorectal cancer tissue showed significantly higher activity of this enzyme compared to the normal mucosa (*p* < 0.01) (Figure 2A). Similarly, xanthine oxidase (XO) activity was higher in tumour than in normal tissue (*p* = 0.01) (Figure 2 B). NOX activity was higher in patients with adenocarcinoma than in adenocarcinoma mucinosum (Figure 2C). Moreover, XO activity was increased in tumour with medium and strong inflammatory infiltration than in absent and weak (Figure 2D). We also observed significantly higher XO activity in CRC patients without vascular invasion in comparison to those with vascular invasion (*p* < 0.05) (Figure 2E).

### 3.3. Antioxidant Defence

**Figure 3:**
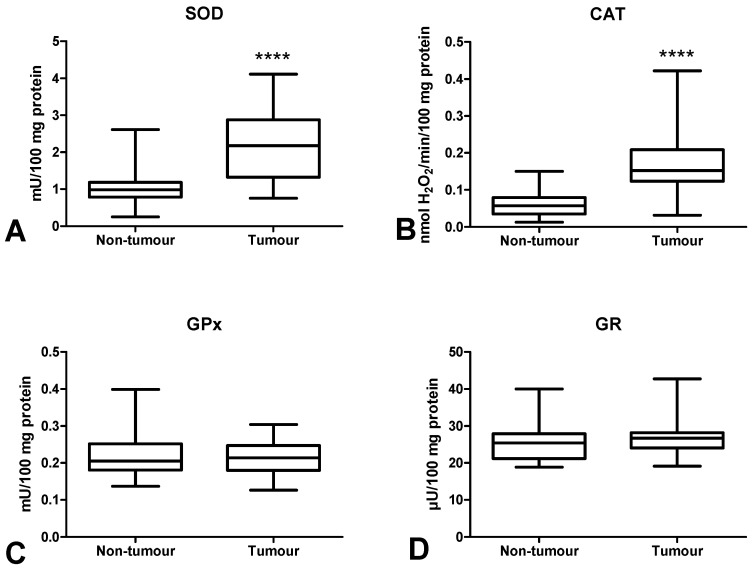
Comparison of SOD (**A**), CAT (**B**), GPx (**C**) and GR (**D**) activity between colorectal cancer tissue (*n* = 29) and normal mucosa (*n* = 29). The data are presented as median (minimum–maximum). Abbreviations: SOD—superoxide dismutase-1, CAT—catalase, GPx—glutathione peroxidase, GR—glutathione reductase, **** *p* < 0.0001.

We measured the activity of antioxidant Cu,Zn-superoxide dismutase (SOD), catalase (CAT), glutathione peroxidase (GPx), glutathione reductase (GR), and concentration of a non-enzymatic antioxidant (reduced glutathione (GSH)) in order to assess the antioxidant barrier. We demonstrated significantly higher SOD and CAT activities in colorectal cancer tissue compared to normal mucosa (*p* < 0.0001, *p* < 0.0001, respectively) (Figure 3A,B). The activities of GPx and GR were slightly higher in tumour tissue than in normal tissue, and differences in the activities of the said enzymes were not statistically significant (Figure 3C,D).

**Figure 4:**
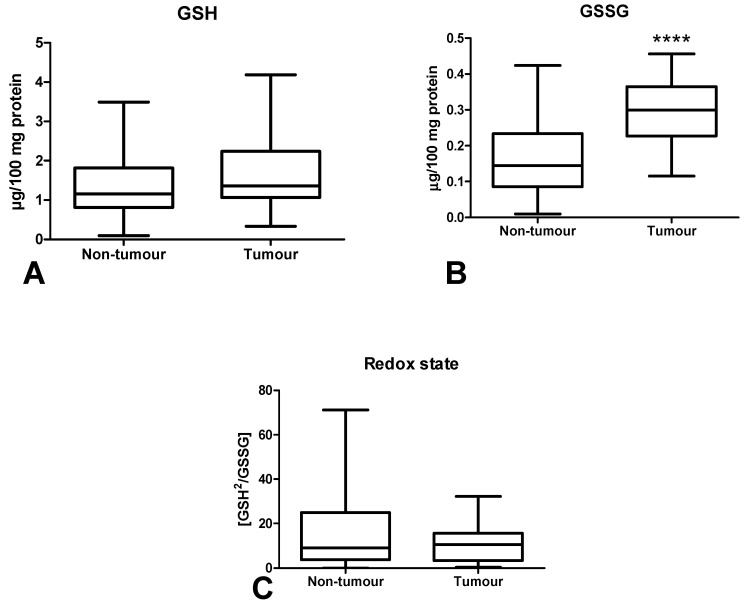
Comparison of GSH (**A**) and GSSG (**B**) concentration and redox state (**C**) between colorectal cancer tissue (*n* = 29) and normal mucosa (*n* = 29). Abbreviations: GSH—reduced glutathione, GSSG—oxidized glutathione (GSSG). The data are presented as median (minimum–maximum). **** *p* < 0.0001.

The concentration of reduced glutathione did not differ significantly between tumour tissue and healthy tissue (Figure 4A). However, the GSSG concentration was significantly higher in the neoplastic tissue (*p* < 0.0001) (Figure 4B). The redox potential (GSH/GSSG ratio) in normal tissue did not differ from cancer tissue (Figure 4C).

**Figure 5:**
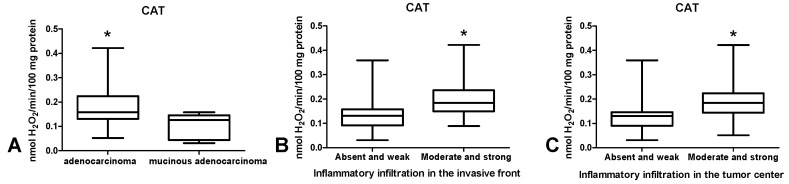
Comparison of: CAT activity (**A**) between adenocarcinoma and mucinous adenocarcinoma; CAT activity depending on the intensity of inflammatory infiltration in the invasive front (**B**) and centre (**C**) of the tumour. The data are presented as median (minimum–maximum). Abbreviations: CAT—catalase. * *p* < 0.05.

CAT activity was significantly higher in tumour tissue in patients with adenocarcinoma compared to those with mucinous adenocarcinoma (*p* < 0.05) (Figure 5A) as well as in patients with inflammatory infiltration both in tumour front and tumour centre, assessed as moderate and strong as opposed to absent and weak (*p* < 0.05, *p* < 0.05) (Figure 5B,C).

### 3.4. Total Antioxidant/Oxidant Status

**Figure 6:**
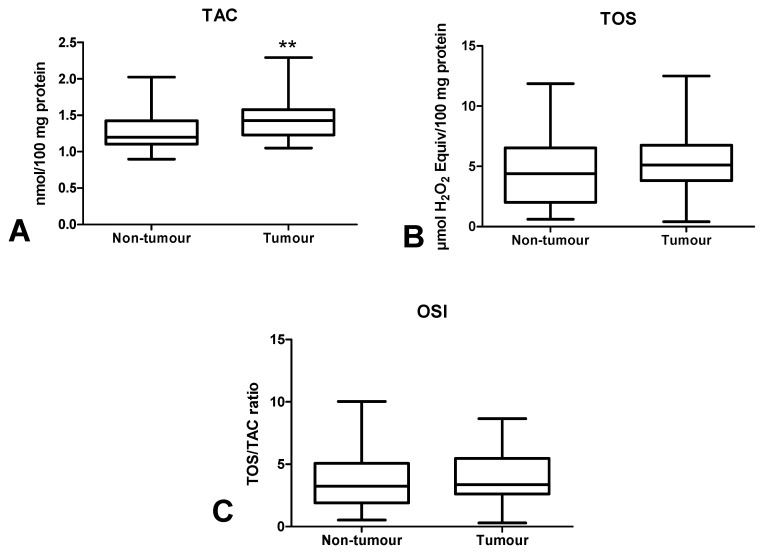
Comparison of TAC (**A**), TOS (**B**) and OSI value (**C**) between colorectal cancer tissue (*n* = 29) and normal mucosa (*n* = 29). The data are presented as median (minimum–maximum). Abbreviations: TAC—total antioxidant capacity, TOS—total oxidant status, OSI—oxidative stress index. ** *p* < 0.01.

The total antioxidant capacity (TAC) and total oxidant status (TOS) are essential for the assessment of redox status, but we also calculated oxidative stress index (OSI), which is the ratio of TOS/TAC. TAC level was significantly higher in the tumour tissue compared to normal mucosa (*p* < 0.01) (Figure 6A). In cancerous tissue, TOS and OSI values were at similar levels to the normal tissue (Figure 6B,C).

**Figure 7:**
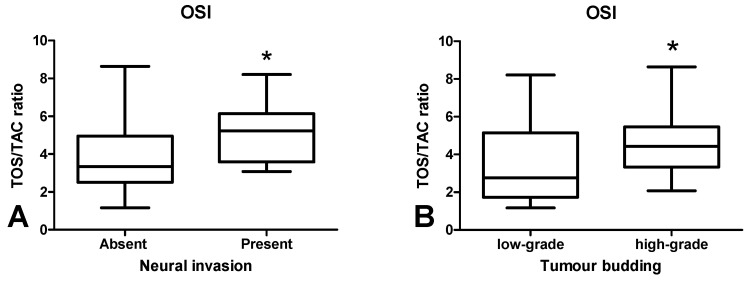
Comparison of OSI value between colorectal tissue with and without neural invasion (**A**) and between colorectal tissue with low-grade and high-grade budding (**B**). The data are presented as median (minimum–maximum). Abbreviations: OSI—oxidative stress index. * *p* < 0.05.

The OSI value was significantly higher in CRC patients with neural invasion in comparison with those without invasion (*p* < 0.05) (Figure 7A). OSI was also higher in patients with tumour budding > 5 than in < 5 (*p* < 0.05) (Figure 7B).

### 3.5. Oxidative Damage to Proteins, Lipids and Nucleic Acids

**Figure 8:**
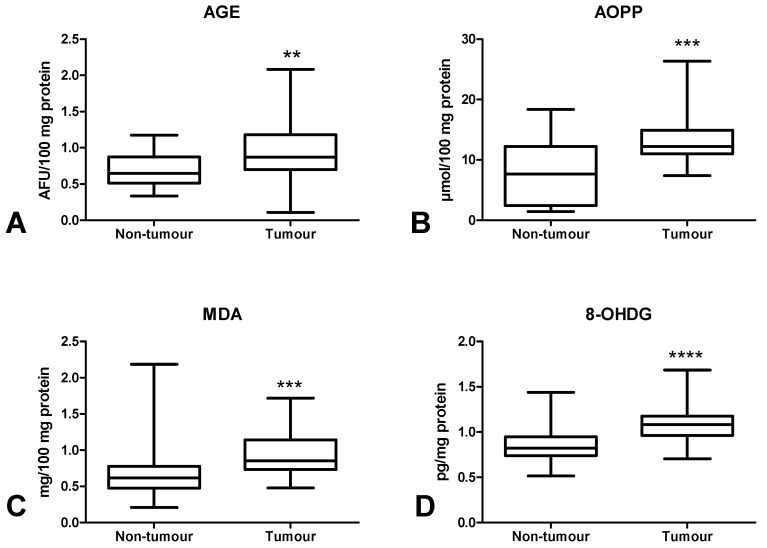
Comparison of AGE (**A**), AOPP (**B**), MDA (**C**) and 8-OHdG (**D**) concentration between colorectal cancer tissue (*n* = 29) and normal mucosa (*n* = 29). The data are presented as median (minimum–maximum). Abbreviations: AGE—advanced glycation end products, AOPP—advanced oxidation protein products, MDA—malondialdehyde, 8-OHdG—8-hydroxydeoxyguanosine. ** *p* < 0.01, *** *p* < 0.001, **** *p* < 0.0001.

**Figure 9:**
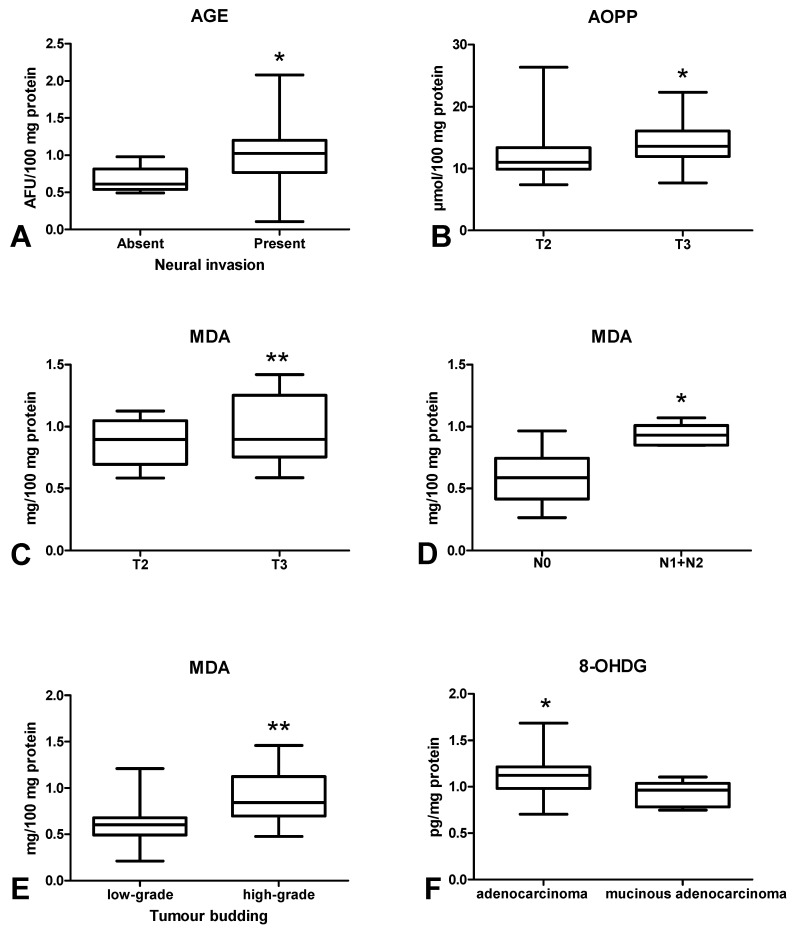
Comparison: of: AGE concentration between colorectal tissue with and without neural invasion (**A**); AOPP (**B**) and MDA (**C**) concentration in colorectal tissue between T2 and T3 stage; MDA concentration between colorectal tissue with and without lymph node metastasis (**D**) as well as between low-grade and high-grade budding (**E**); 8-OHdG concentration (**F**) between adenocarcinoma and mucinous adenocarcinoma. The data are presented as median (minimum–maximum). Abbreviations: AGE—advanced glycation end products, AOPP—advanced oxidation protein products, MDA—malondialdehyde, 8-OHdG—8-hydroxydeoxyguanosine. * *p* < 0.05, ** *p* < 0.01.

In order to assess oxidative stress, we used products of oxidative damage to proteins (i.e., advanced glycation end products (AGE), advanced oxidation protein products (AOPP)), lipids (malondialdehyde (MDA)] and DNA [8-hydroxydeoxyguanosine (8-OHdG)). There was a statistically significant increase in AGE (Figure 8A) and AOPP (Figure 8B) contents in colorectal cancer tissue compared to the normal mucosa (*p* < 0.01, *p* < 0.001). Furthermore, MDA (Figure 8C) and 8-OHdG (Figure 8D) concentrations were significantly higher in the tumour than in normal tissue (*p* < 0.001, *p* < 0.0001, respectively) (Figure 8). AGE concentration was significantly lower in CRC patients without neural invasion than in those with invasion (*p* < 0.05) (Figure 9A). AOPP (Figure 9B) and MDA (Figure 9C) levels were higher in tumour tissue in stage T3 compared to T2 (*p* < 0.05). Moreover, we observed a statistically significant increase in MDA concentration in CRC patients with lymph node metastases compared to subjects without metastases (*p* < 0.05) (Figure 9D) as well as in CRC patients with tumour budding > 5 compared to tumour budding < 5 (*p* < 0.001) (Figure 9E). 8-OHdG level was higher in patients with adenocarcinoma than in those with mucinous adenocarcinoma (*p* < 0.05) (Figure 9F).

### 3.6. Correlations

**Figure 10:**
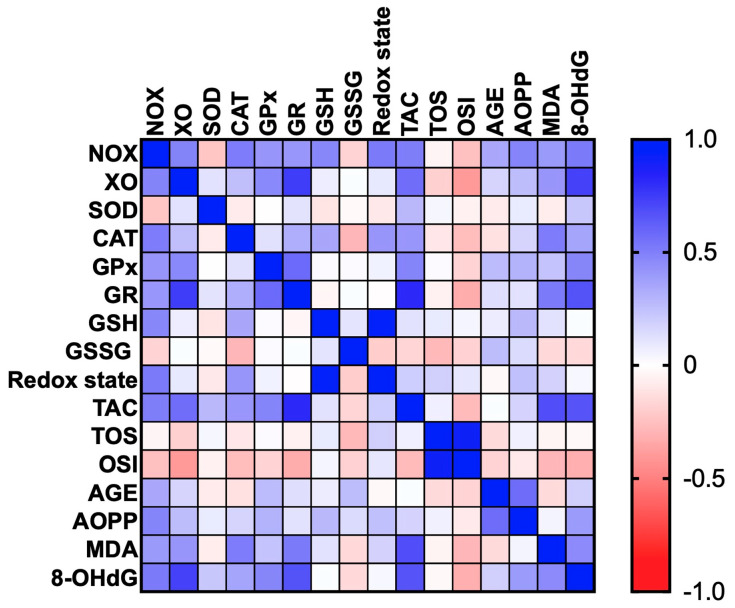
Heat map of oxidative stress parameters in the tumour tissue (*n* = 29).

All statistically significant correlations are presented in Figure 10. Interestingly, we observed a positive correlation between NOX and CAT (*p* < 0.005, R = 0.509), NOX and TAC (*p* < 0.005, R = 0.504) and NOX and 8-OHdG (*p* < 0.005, R = 0.525). We also demonstrated correlation between XO and GR (*p* < 0.0001, R = 0.745), XO and TAC (*p* = 0.001, R = 0.568), and XO and 8-OHdG (*p* < 0.0001, R = 0.731) as well as between GPx and GR (*p* < 0.001, R = 0.582), GPx and TAC (*p* = 0.009, R = 0.477), and GPx and 8-OHdG (*p* = 0.009, R = 0.474). Moreover, statistically significant correlations were found between GR and TAC (*p* < 0.0001, R = 0.818), GR and MDA (*p* < 0.005, R = 0.516), and GR and 8-OHdG (*p* < 0.001, R = 0.671). We also observed positive correlations between TAC and MDA (*p* < 0.0001, R = 0.685) and TAC and 8-OHdG (*p* < 0.0001, R = 0.661). TOS value was positively correlated with OSI (*p* < 0.0001, R = 0.912). Importantly, we showed a positive correlation between AGE and AOPP (*p* < 0.001, R = 0.571).

## 4. Discussion

In our research, we evaluated selected biomarkers of redox homeostasis in colorectal cancer and normal adjacent tissues. The presented study is the first to assess antioxidant barrier, redox status, and oxidative damage in respect of histopathological parameters associated with tumour microenvironment, such as tumour budding and inflammatory infiltration. We demonstrated differences in chosen enzymatic and non-enzymatic antioxidants as well as enhanced oxidation of proteins, lipids and DNA in colorectal cancer tissue compared to normal tissue. Moreover, we observed a link between XO/CAT activity and inflammatory infiltration in the front and CAT activity in the centre of the tumour as well as between OSI and MDA and tumour budding, which may suggest the participation of oxidative stress in the remodelling of tumour stroma. Additionally, we observed significantly higher MDA level in patients with lymph node metastasis in comparison to those without metastasis.

Oxidative stress may be associated with the development of different systemic diseases such as obesity [27], diabetes, insulin resistance [10], genetic conditions [28], neurodegenerative disorders [29], and cardiovascular diseases [30], as well as in some types of cancer, e.g., gastric cancer [31], breast cancer [32] and melanoma [33]. ROS overproduction together with insufficient antioxidant defence lead to oxidative cell damage, which promotes the initiation/progression of cancer. In living organisms, the main source of ROS is respiratory transport chain generating O_2_^−^ [34]. However, apart from mitochondrial respiration, another source of ROS are pro-oxidant enzymes such as NOX and XO. NADPH oxidase is a multi-subunit complex generating O_2_^−^ and H_2_O_2_ [35]. It has been demonstrated that neutrophils and macrophages produce more ROS (via NOX activity) than vascular smooth muscles and endothelial cells. However, NOX is also a very important ROS source in cancer cells and it generates high amounts of pro-inflammatory cytokines. Indeed, increased activity of NOX creates pro-oxidative environment, thus promoting genomic instability and tissue remodelling in tumour tissues [36]. The second pro-oxidant enzyme, XO, participates in hypoxanthine and xanthine conversion into uric acid. As a by-product, XO also generates superoxide radicals and hydrogen peroxide [37]. Additionally, XO increases HIF-1α expression and activates NF-κB pathway, which promotes inflammation and cancer progression [38,39]. However, pro-inflammatory cytokines also affect the production of free radicals. It was shown that IL-1α, IL-1β, IL-6, IL-8 and vascular endothelial growth factor (VEGF) induce the secondary production of ROS through a positive feedback mechanism (by stimulating NOX and XO activity) [40]. In our study, we observed enhanced activity of NOX and XO in colon cancer tissue. Simultaneously, we demonstrated higher NOX activity in adenocarcinoma patients compared to those with mucinous adenocarcinoma, as well as higher XO activity in moderate and strong inflammatory infiltration in comparison with absent and weak infiltration. Thus, enhanced activity of pro-oxidant enzymes may intensify inflammatory processes and lead to malignant transformation. Indeed, these proteins may induce changes in the phenotype of cancer cells, influencing the processes of their proliferation and angiogenesis or enabling tumour escape from immune surveillance [36]. Additionally, studies performed on cell lines showed that the decreased expression of NOX1 inhibits cell division in vascular smooth muscle [41]. Furthermore, the suppression of NOX5 in Barrett’s adenocarcinoma cells hinders proliferation and growth potential [41]. Therefore, it is not surprising that NOX overexpression may influence the rate of cell division.

We also assessed the content of antioxidant enzymes and observed increased activity of CAT and SOD in tumour tissue compared to normal tissue. This suggests an adaptive response to the overproduction of intracellular ROS in cancer cells [42]. However, increased SOD activity also leads to enhanced formation of hydrogen peroxide in colon tissue. This was confirmed by Szatrowski et al., who demonstrated that CRC is accompanied by the production of a large amount of H_2_O_2_ [43]. Nevertheless, hydrogen peroxide can be decomposed by catalase or glutathione peroxidase. Catalase participates in the conversion of hydrogen peroxide at a high ROS level [44]. While ROS production is low, GPx reveals a much higher affinity to H_2_O_2_. This may be explained by differences in the Michaelis–Menten constant (Km) of CAT and GPx. The Km for CAT (Km = 2.4 × 10^−4^ M) is significantly higher than the Km for GPx (Km = 1 × 10^−6^ M), which indicates that CAT scavenges H_2_O_2_ efficiently at higher concentrations [45]. Therefore, although we did not directly assess the rate of free radical production, our results suggest enhanced ROS levels in CRC patients. Simultaneously, we observed significantly higher CAT activity in patients with adenocarcinoma than in subjects suffering from mucinous adenocarcinoma. It should be remembered that mucinous adenocarcinoma is commonly considered a more aggressive type of CRC. CAT activity was also higher in patients with moderate and strong inflammatory infiltration both in the front of the tumour and in tumour centre than in weak infiltration. This is not surprising because inflammatory cells such as neutrophils and macrophages are characterized by enhanced activity of superoxide dismutase, which leads to increased production of hydrogen peroxide. Pietarinen-Runtti et al. [45] observed that among all inflammatory cells, CAT activity is the highest in neutrophils. It is suggested that CAT may play a profound role in the proper phagocytosis during oxidative stress of activated neutrophils.

It is difficult to assess the redox status based solely on the level of individual antioxidants. Therefore, we evaluated the biomarkers characterizing the resultant antioxidant/oxidant capacity (TAC, TOS, OSI) in colorectal cancer patients. TAC describes the sum of enzymatic and non-enzymatic antioxidants, whereas TOS allows for the assessment of all oxidants contained in a sample. OSI (TOS/TAC ratio) indicates the relationship between antioxidant mechanisms and oxidant concentrations [46]. In our work, we observed considerably higher TAC levels in cancerous tissue compared to normal tissue, whereas TOS and OSI contents did not differ significantly between both groups. This confirms the previous hypothesis of an antioxidant adaptive response to ROS overproduction in CRC patients, as also evidenced by the positive correlation between NOX and CAT (*p* < 0.005, R = 0.509), NOX and TAC (*p* < 0.005, R = 0.504) and XO and GR (*p* < 0.0001, R = 0.745). However, ROS in the colon may not only originate from the pro-oxidant enzymes, but also diet and gut microflora. Interestingly, human gut microflora contains up to 500 bacteria species generating oxygen free radicals [4]. To assess the redox status, we also used the ratio of reduced glutathione (GSH) to oxidized glutathione (GSSG). The oxido-reductive potential did not differentiate the studied groups, despite the fact that the GSSG concentration was significantly higher in the cancer tissue. Nevertheless, accumulation of GSSG in a cell can be cytotoxic. In the long term, GSH reserves may also be exhausted. Since only patients with G2 grade were involved in our investigation, it is necessary to carry out further studies on cases with different tumour differentiation grade. Interestingly, OSI level was also increased in patients with neural invasion compared to those without invasion. Moreover, it was higher in patients with high-grade budding than five versus cases with low-grade budding. Therefore, OSI may be an unfavourable prognostic factor because neural invasion is one of the indicators of tumour advancement. It is well known that high-grade tumour budding is strongly associated with shorter cancer-specific survival [47]. However, it should be remembered that OSI does not differentiate between tumour tissue and healthy tissue.

Oxidative stress disrupts cell metabolism through oxidative damage to its components (e.g., proteins, lipids and DNA). The most commonly evaluated biomarkers of protein glyco-oxidation are advanced glycation end products (AGEs) and advanced oxidation protein products (AOPPs). AOPPs are crosslinked protein adducts formed as a result of the action of chlorinated oxidants (mainly hypochlorous acid and chloramines) which are generated by myeloperoxidase in activated neutrophils [48]. It has been observed that AOPPs may act as inflammatory mediators causing the oxidative ignition of neutrophils, monocytes, and T lymphocytes [48]. AGEs and AOPPs bind to receptors for advanced glycation end products (RAGE), stimulating intracellular formation of ROS as well as several transcription factors (e.g., NF-kB, MAP-kinase, NJK and p21RAS) [49]. In this study, both AGE and AOPP levels were significantly higher in tumour tissue compared to normal colon mucosa. It is suggested that the accumulation of oxidative protein products is responsible for chronic inflammation and enhanced production of ROS. Indeed, AOPP and AGE can accumulate and aggregate in cancer tissue, which enhances NOX activity and stimulates (through positive feedback) NF-kB expression [50]. This results in the development of an inflammatory microenvironment which contributes to tumour onset by promoting genetic instability, cell survival, growth and metastatic potential [51]. As this goes beyond the framework of our study, it is necessary to assess the pro-inflammatory cytokines/chemokines in respect to histopathological parameters associated with the tumour microenvironment. In our experiment, we also observed significantly higher AGE level in cancerous tissue in patients with neural invasion. It is commonly known that neural invasion has been recognized as a poor prognostic factor in several malignancies. Therefore, increased AGE content (similar to OSI) may be an unfavourable prognostic factor portending the poor prognosis for patients. Additionally, AOPP level was higher in tumours penetrating subserosa (T3) than in tumours restricted to the muscularis propria (T2). Consequently, protein oxidation in colorectal cancer is enhanced with increased depth of tumour invasion.

The most effective indicator and simultaneously the most harmful product of lipid peroxidation is malondialdehyde. Indeed, MDA is responsible for changes in gene expression, genetic mutations, molecular heterogeneity as well as impairment of intercellular communication [52]. In this study, MDA level was lower in tumours penetrating the muscularis propria (T2) than in tumours penetrating subserosa (T3). Therefore, lipid peroxidation is enhanced with the increase in the depth of tumour invasion. It is suggested that MDA can act as a carcinogenic agent and promoter of CRC development/progression [53]. This is also confirmed by higher MDA level in patients with high-grade tumour budding as well as in subjects with lymph node metastases. Malondialdehyde can easily pass from the sites of its formation into remote tissues and form covalent bonds to proteins and nucleic acids. Therefore, MDA may modify the structure and functional properties of cells. Our results suggest that MDA cytotoxicity increases with CRC advancement. MDA can participate in malignant transformation connected with the depth of colon wall invasion, tumour budding and metastasis. However, in our work, we also assessed 8-hydroxydeoxyguanosine, which is the indicator of oxidative DNA damage. 8-OHdG is the most abundant and stable product of DNA oxidative injury [54]. Interestingly, 8-OHdG level was significantly higher in cancerous tissue than in normal colon mucosa. Therefore, DNA damage repair systems are less efficient in CRC patients. It is well known that DNA integrity and stability are a prerequisite for the proper functioning of cells. Oxidative modifications of nucleic acids not only initiate the neoplastic process, but also promote the transformation of benign lesions into malignant ones. They may also increase the metastatic potential.

## 5. Conclusions

In conclusion, CRC development is associated with enzymatic and non-enzymatic redox imbalance as well as increased oxidative damage to proteins, lipids and DNA. We also observed differences in oxidative stress parameters between different stages of CRC advancement. The determination of these parameters could be useful for the evaluation of the tumour progression. We also showed that redox indicators may be engaged in inflammatory infiltration and tumour budding. These parameters are associated with the tumour microenvironment. Moreover, they are considered independent adverse prognostic factors in patients with primary operable colorectal cancer. Therefore, the assessment of oxidative stress biomarkers may be useful in CRC patient’s prognosis. However, further studies are required on a larger group of patients.

It is also important to note certain limitations of our research. We evaluated only the selected redox biomarkers, so we are not able to fully characterize the oxido-reductive balance of patients with colorectal cancer. Due to the small amount of research material, we were also unable to assess certain kinases and transcription factors that participate in progressive cancer growth. In addition, the tumour differentiation grade in all patients was G2. In the future, it would be interesting and advisable to assess the relationship between the intensity of oxidative stress and the survival rate of CRC patients. It is also necessary to perform molecular analyses to explain oxidative stress-related pathways.

## Figures and Tables

**Table 1:** Characteristics of the study group (*n* = 29).

Parameter	*n* (%)
**Age**	
<60	5 (17.2%)
>60	24 (82.8%)
**Gender**	
male	16 (55.2%)
female	13 (44.8%)
**Location**	
sigmoid colon	7 (24.2%)
rectum	8 (27.6%)
cecum	4 (13.8%)
ascending colon	3 (10.3%)
hepatic fold	4 (13.8%)
colon	3 (10.3%)
**Tumour size**	
<3 cm	5 (17.2%)
>3 cm	24 (82.8%)
**Histological type**	
adenocarcinoma	25 (86.2%)
mucinous adenocarcinoma	4 (13.8%)
**Depth of tumour invasion (pT)**	
T1	0 (0.0%)
T2	10 (34.5%)
T3	19 (65.5%)
T4	0 (0.0%)
**Lymph node metastasis (pN)**	
N0	18 (62.1%)
N1+N2	11 (37.9%)
**Distant metastasis (pM)**	
M0	26 (89.7%)
M1	3 (10.3%)
**Stage at diagnosis**	
I	8 (27.6%)
II	7 (24.1%)
III	10 (34.5%)
IV	4 (13.8%)
**Vascular invasion**	
absent	12 (41.4%)
present	17 (58.6%)
**Neural invasion**	
absent	21 (72.4%)
present	8 (27.6%)
**Inflammatory infiltration in the invasive front**	
absent	0 (0.0%)
weak	14 (48.3%)
moderate	11 (37.9%)
strong	4 (13.8%)
**Inflammatory infiltration in the tumour centre**	
absent	1 (34.5%)
weak	15 (51.7%)
moderate	11 (37.9%)
strong	2 (6.9%)
**Tumour budding (TB)**	
1 – <5	2 (6.9%)
2 – 5–9	10 (34.5%)
3 – >10	8 (27.6%)
	9 (31.0%)
**Poorly differentiated clusters (PDC)**	
1 – <5	0 (0.0%)
2 – 5–9	29 (100.0%)
3 – >10	0 (0.0%)
	0 (0.0%)
**Areas of poorly differentiated components (APDC)**	
0	0 (0.0%)
1 – <10	29 (100.0%)
2 – >10	0 (0.0%)

## Supplementary Materials

The following are available online at https://www.mdpi.com/2072-6694/12/6/1636/s1, Table S1: Oxidative stress biomarkers in relation to clinical-pathological parameters, Table S2: Comparison of oxidative stress biomarkers between tumour tissue and normal adjacent mucosa.

## References

[1] Ferlay J., Ervik M., Lam F., Colombet M., Mery L., Piñeros M., Znaor A., Soerjomataram I.B.F. (2018). Global Cancer Observatory: Cancer Today.

[2] Adam R., de Gramont A., Figueras J., Kokudo N., Kunstlinger F., Loyer E., Poston G., Rougier P., Rubbia-Brandt L., Sobrero A. (2015). Managing synchronous liver metastases from colorectal cancer: A multidisciplinary international consensus. Cancer Treat. Rev..

[3] Fiaschi T., Chiarugi P. (2012). Oxidative Stress, Tumor Microenvironment, and Metabolic Reprogramming: A Diabolic Liaison. Int. J. Cell Biol..

[4] Zińczuk J., Maciejczyk M., Zaręba K., Romaniuk W., Markowski A., Kędra B., Zalewska A., Pryczynicz A., Matowicka-Karna J., Guzińska-Ustymowicz K. (2019). Antioxidant Barrier, Redox Status, and Oxidative Damage to Biomolecules in Patients with Colorectal Cancer. Can Malondialdehyde and Catalase Be Markers of Colorectal Cancer Advancement?. Biomolecules.

[5] Bosman F., Carneiro F., Hruban R., Theise N., Intergovernmental Panel on Climate Change (2010). Summary for Policymakers. Climate Change 2013—The Physical Science Basis.

[6] Amin M.B. (2017). AJCC Cancer Staging System.

[7] Jass J.R., Sobin L.H. (1989). Histological Typing of Intestinal Tumours.

[8] Ueno H., Kajiwara Y., Shimazaki H., Shinto E., Hashiguchi Y., Nakanishi K., Maekawa K., Katsurada Y., Nakamura T., Mochizuki H. (2012). New criteria for histologic grading of colorectal cancer. Am. J. Surg. Pathol..

[9] Zalewska A., Knaś M., Zendzian-Piotrowska M., Waszkiewicz N., Szulimowska J., Prokopiuk S., Waszkiel D., Car H. (2014). Antioxidant profile of salivary glands in high fat diet-induced insulin resistance rats. Oral Dis..

[10] Kolodziej U., Maciejczyk M., Miasko A., Matczuk J., Knas M., Zukowski P., Zendzian-Piotrowska M., Borys J., Zalewska A. (2017). Oxidative modification in the salivary glands of high fat-diet induced insulin resistant rats. Front. Physiol..

[11] Walker J.M. (1994). The bicinchoninic acid (BCA) assay for protein quantitation. Methods Mol. Biol..

[12] Griendling K.K., Minieri C.A., Ollerenshaw J.D., Alexander R.W. (1994). Angiotensin II stimulates NADH and NADPH oxidase activity in cultured vascular smooth muscle cells. Circ. Res..

[13] Prajda N., Weber G. (1975). Malignant transformation-linked imbalance: Decreased xanthine oxidase activity in hepatomas. FEBS Lett..

[14] Aebi H. (1984). [13] Catalase in Vitro. Methods Enzymol..

[15] Paglia D.E., Valentine W.N. (1967). Studies on the quantitative and qualitative characterization of erythrocyte glutathione peroxidase. J. Lab. Clin. Med..

[16] Mize C.E., Langdon R.G. (1962). Hepatic glutathione reductase. I. Purification and general kinetic properties. J. Biol. Chem..

[17] Misra H.P., Fridovich I. (1972). The role of superoxide anion in the autoxidation of epinephrine and a simple assay for superoxide dismutase. J. Biol. Chem..

[18] Griffith O.W. (1980). Determination of glutathione and glutathione disulfide using glutathione reductase and 2-vinylpyridine. Anal. Biochem..

[19] Zalewska A., Ziembicka D., Zendzian-Piotrowska M., MacIejczyk M. (2019). The impact of high-fat diet on mitochondrial function, free radical production, and nitrosative stress in the salivary glands of wistar rats. Oxid. Med. Cell. Longev..

[20] Erel O. (2004). A novel automated direct measurement method for total antioxidant capacity using a new generation, more stable ABTS radical cation. Clin. Biochem..

[21] Erel O. (2005). A new automated colorimetric method for measuring total oxidant status. Clin. Biochem..

[22] Knaś M., Maciejczyk M., Daniszewska I., Klimiuk A., Matczuk J., Kołodziej U., Waszkiel D., Ładny J.R., Zendzian-Piotrowska M., Zalewska A. (2016). Oxidative Damage to the Salivary Glands of Rats with Streptozotocin-Induced Diabetes-Temporal Study: Oxidative Stress and Diabetic Salivary Glands. J. Diabetes Res..

[23] Kalousová M., Škrha J., Zima T. (2002). Advanced glycation end-products and advanced oxidation protein products in patients with diabetes mellitus. Physiol. Res..

[24] Buege J.A., Aust S.D. (1978). Microsomal Lipid Peroxidation. Methods Enzymol..

[25] Loughrey M., Quirke P., Shepherd N.A.N., Hospital G.R. (2017). Standards and Datasets for Reporting Cancers.

[26] Liebig C., Ayala G., Wilks J.A., Berger D.H., Albo D. (2009). Perineural invasion in cancer: A review of the literature. Cancer.

[27] Fejfer K., Buczko P., Niczyporuk M., Ładny J.R., Hady H.R., Knaś M., Waszkiel D., Klimiuk A., Zalewska A., Maciejczyk M. (2017). Oxidative Modification of Biomolecules in the Nonstimulated and Stimulated Saliva of Patients with Morbid Obesity Treated with Bariatric Surgery. Biomed. Res. Int..

[28] Maciejczyk M., Heropolitanska-Pliszka E., Pietrucha B., Sawicka-Powierza J., Bernatowska E., Wolska-Kusnierz B., Pac M., Car H., Zalewska A., Mikoluc B. (2019). Antioxidant Defense, Redox Homeostasis, and Oxidative Damage in Children With Ataxia Telangiectasia and Nijmegen Breakage Syndrome. Front. Immunol..

[29] Klimiuk A., Maciejczyk M., Choromańska M., Fejfer K., Waszkiewicz N., Zalewska A. (2019). Salivary Redox Biomarkers in Different Stages of Dementia Severity. J. Clin. Med..

[30] Touyz R.M. (2004). Reactive Oxygen Species, Vascular Oxidative Stress, and Redox Signaling in Hypertension. Hypertension.

[31] Borrego S., Vazquez A., Dasí F., Cerdá C., Iradi A., Tormos C., Sánchez J., Bagán L., Boix J., Zaragoza C. (2013). Oxidative Stress and DNA Damage in Human Gastric Carcinoma: 8-Oxo-7‘8-dihydro-2’-deoxyguanosine (8-oxo-dG) as a Possible Tumor Marker. Int. J. Mol. Sci..

[32] Sawczuk B., Maciejczyk M., Sawczuk-Siemieniuk M., Posmyk R., Zalewska A., Car H. (2019). Salivary Gland Function, Antioxidant Defence and Oxidative Damage in the Saliva of Patients with Breast Cancer: Does the BRCA1 Mutation Disturb the Salivary Redox Profile?. Cancers (Basel).

[33] Obrador E., Liu-Smith F., Dellinger R.W., Salvador R., Meyskens F.L., Estrela J.M. (2019). Oxidative stress and antioxidants in the pathophysiology of malignant melanoma. Biol. Chem..

[34] Kang J., Pervaiz S. (2012). Mitochondria: Redox metabolism and dysfunction. Biochem. Res. Int..

[35] Meitzler J.L., Konaté M.M., Doroshow J.H. (2019). Hydrogen peroxide-producing NADPH oxidases and the promotion of migratory phenotypes in cancer. Arch. Biochem. Biophys..

[36] Block K., Gorin Y. (2012). Aiding and abetting roles of NOX oxidases in cellular transformation. Nat. Rev. Cancer.

[37] Battelli M.G., Musiani S., Valgimigli M., Gramantieri L., Tomassoni F., Bolondi L., Stirpe F. (2001). Serum xanthine oxidase in human liver disease. Am. J. Gastroenterol..

[38] Romagnoli M., Gomez-Cabrera M.C., Perrelli M.G., Biasi F., Pallardó F.V., Sastre J., Poli G., Viña J. (2010). Xanthine oxidase-induced oxidative stress causes activation of NF-κB and inflammation in the liver of type I diabetic rats. Free Radic. Biol. Med..

[39] Balamurugan K. (2016). HIF-1 at the crossroads of hypoxia, inflammation, and cancer. Int. J. Cancer.

[40] Maciejczyk M., Szulimowska J., Taranta-janusz K., Wasilewska A., Zalewska A. (2020). Salivary Gland Dysfunction, Protein Glycooxidation and Nitrosative Stress in Children with Chronic Kidney Disease. J. Clin. Med..

[41] Lambeth J.D. (2007). Nox enzymes, ROS, and chronic disease: An example of antagonistic pleiotropy. Free Radic. Biol. Med..

[42] Lushchak V.I. (2014). Free radicals, reactive oxygen species, oxidative stress and its classification. Chem. Biol. Interact..

[43] Szatrowski T.P., Nathan C.F. (1991). Production of Large Amounts of Hydrogen Peroxide by Human Tumor Cells. Cancer Res..

[44] Kirkman H.N., Gaetani G.F. (2007). Mammalian catalase: A venerable enzyme with new mysteries. Trends Biochem. Sci..

[45] Hetarinen-Runtti P., Lakari E., Raivio K.O., Kinnula V.L. (2000). Expression of antioxidant enzymes in human inflammatory cells. Am. J. Physiol. Cell Physiol..

[46] Skutnik-Radziszewska A., Maciejczyk M., Fejfer K., Krahel J., Flisiak I., Kołodziej U., Zalewska A. (2020). Salivary Antioxidants and Oxidative Stress in Psoriatic Patients: Can Salivary Total Oxidant Status and Oxidative Status Index Be a Plaque Psoriasis Biomarker?. Oxid. Med. Cell. Longev..

[47] Van Wyk H.C., Park J.H., Edwards J., Horgan P.G., McMillan D.C., Going J.J. (2016). The relationship between tumour budding, the tumour microenvironment and survival in patients with primary operable colorectal cancer. Br. J. Cancer.

[48] Alderman C.J.J., Shah S., Foreman J.C., Chain B.M., Katz D.R. (2002). The role of advanced oxidation protein products in regulation of dendritic cell function. Free Radic. Biol. Med..

[49] Neumann A., Schinzel R., Palm D., Riederer P., Münch G. (1999). High molecular weight hyaluronic acid inhibits advanced glycation endproduct-induced NF-κB activation and cytokine expression. FEBS Lett..

[50] Ott C., Jacobs K., Haucke E., Navarrete Santos A., Grune T., Simm A. (2014). Role of advanced glycation end products in cellular signaling. Redox Biol..

[51] Turner D.P. (2015). Advanced glycation end-products: A biological consequence of lifestyle contributing to cancer disparity. Cancer Res..

[52] Całyniuk B., Grochowska-Niedworok E., Walkiewicz K., Kawecka S., Popiołek E., Fatyga E. (2016). Malondialdehyde (MDA)—Product of lipid peroxidation as marker of homeostasis disorders and aging. Ann. Acad. Med. Silesiensis.

[53] Rašić I., Rašić A., Akšamija G., Radović S. (2018). The relationship between serum level of malondialdehyde and progression of colorectal cancer. Acta Clin. Croat..

[54] Fenga C., Gangemi S., Teodoro M., Rapisarda V., Golokhvast K., Docea A.O., Tsatsakis A.M., Costa C. (2017). 8-Hydroxydeoxyguanosine as a biomarker of oxidative DNA damage in workers exposed to low-dose benzene. Toxicol. Rep..

