# Supplementary material for: Pro-Oxidant Enzymes, Redox Balance and Oxidative Damage to Proteins, Lipids and DNA in Colorectal Cancer Tissue. Is Oxidative Stress Dependent on Tumour Budding and Inflammatory Infiltration?
Source: Cancers (Basel). 2020 Jun 20;12(6):1636. doi: 10.3390/cancers12061636 (PMC7352177; doi:10.3390/cancers12061636)
Supplement: Supplementary file 1 [file cancers-12-01636-s001.pdf]

Supplementary Materials: Pro-Oxidant Enzymes, Redox Balance and Oxidative Damage to Proteins, Lipids and DNA in Colorectal Cancer Tissue. Is Oxidative Stress Dependent on Tumour Budding and Inflammatory Infiltration?

Justyna Zińczuk, Mateusz Maciejczyk, Konrad Zaręba, Anna Pryczynicz, Violetta Dymicka-Piekarska, Joanna Kamińska, Olga Koper-Lenkiewicz, Joanna Matowicka-Karna, Bogusław Kędra, Anna Zalewska and Katarzyna Guzińska-Ustymowicz

Table S1. Oxidative stress biomarkers in relation to clinical-pathological parameters.

Parameter	Male vs female		adenocarcinoma vs mucinous adenocarcinoma		T2 vs T3 (depth of invasion)		N0 vs N1+N2 (lymph node metastasis)		M0 vs M1 (distant metastasis)		Stage at diagnosis - I+II vs III+IV		Absent vs present vascular invasion	Absent vs present neural invasion	Inflammatory infiltration in the invasive front: absent and weak vs moderate and strong	Inflammatory infiltration in the tumor center: absent and weak vs moderate and strong	Tumour budding <5 vs tumour budding >5
	**Non-tumour**	**Tumour**	**Non-tumour**	**Tumour**	**Non-tumour**	**Tumour**	**Non-tumour**	**Tumour**	**Non-tumour**	**Tumour**	**Non-tumour**	**Tumour**	**Tumour**	**Tumour**	**Tumour**	**Tumour**	**Tumour**
**NOX**	p=0.121 4.032 (2.179-5.236) vs 4.925 (1.660-19.79)	p=0.242 5.355 (5.165-9.165) vs 6.414 (4.941-12.06)	p=0.544 5.01 (1.79-17.66) vs 4.54 (3.52-9.31)	**p<0.05** 6.60 (5.03-11.73) vs 5.24 (4.94-9.57)	p=0.477 4.93 (1.66-9.54) vs 4.77 (3.15-19.79)	p=0.303 5.56 (5.17-9.17) vs 6.41 (4.94-12.06)	p=0.840 4.84 (1.66-19.79) vs 4.77 (3.15-11.26)	p=0.702 5.79 (4.98-12.06) vs 6.41 (4.94-10.75)	p=0.802 4.85 (2.18-11.27) vs 4.76 (4.41-8.16)	p=0.333 6.04 (4.97-10.75) vs 8.36 (5.51-10.12)	p=0.889 4.925 (1.660-19.79) vs 4.767 (3.152-11.26)	p=0.389 5.613 (4.980-10.72) vs 6.271 (5.456-10.11)	p=0.550 5.78 (4.94-12.05) vs 6.41 (5.16-10.71)	p=0.296 6.89 (4.98-10.75) vs 5.86 (5.16-7.49)	p=0.092 6.89 (5.46-12.06) vs 5.47 (4.94-10.75)	p=0.196 6.89 (4.98-12.06) vs 5.51 (4.94-10.72)	p=0.278 7.64 (4.98-12.05) vs 5.96 (4.94-10.72)
**XO**	p=0.297 10.98 (10.20-12.40) vs 12.10 (9.47-18.84)	***p=0.039*** 10.88 (10.19-14.17) vs 13.56 (10.17-21.05)	p=0.665 12.11 (9.48-18.46) vs 11.96 (10.20-13.05)	p=0.126 13.86 (10.18-20.28) vs 12.69 (10.53-14.07)	p=0.396 11.69 (9.59-14.73) vs 12.10 (9.47-18.84)	p=0.176 12.69 (10.15-17.88) vs 14.07 (10.07-21.16)	p=0.357 11.84 (9.47-18.84) vs 12.59 (9.54-14.73)	p=0.946 13.54 (10.19-17.96) vs 13.19 (10.17-21.05)	p=0.802 12.05 (9.54-17.35) vs 11.95 (11.72-13.16)	p=0.957 13.54 (10.19-17.96) vs 13.30 (12.69-14.88)	p=0.718 12.01 (9.47-18.84) vs 12.15 (9.54-14.73)	p=0.454 13.56 (10.19-17.96) vs 12.78 (10.17-14.88)	***p<0.05*** 14.30 (11.23-21.05) vs 12.87 (10.17-16.25)	p=0.296 13.54 (11.23-17.96) vs 12.69 (10.17-16.25)	***p<0.05*** 12.69 (10.19-21.05) vs 14.41 (10.17-17.96)	p=0.346 13.91 (10.17-21.05) vs 13.30 (10.19-15.34)	p=0.549 13.79 (11.23-21.05) vs 13.30 (10.17-16.47)
**SOD**	p=0.681 1.010 (0.835-1.272) vs 0.986 (0.254-2.608)	p=0.658 1.932 (0.939-2.960) vs 2.178 (0.751-4.112)	p=0.751 1.01 (0.29-2.34) vs 0.94 (0.83-1.08)	p=0.654 2.21 (0.76-4.00) vs 1.35 (0.94-3.97)	p=0.836 0.96 (0.47-1.27) vs 1.00 (0.25-2.61)	p=0.251 1.49 (0.94-2.96) vs 2.24 (0.75-4.11)	p=0.458 0.99 (0.47-1.45) vs 0.84 (0.25-2.61)	p=0.164 1.61 (0.75-3.68) vs 2.38 (0.93-4.11)	p=0.092 0.93 (0.44-1.45) vs 1.30 (0.99-2.61)	p=0.142 2.21 (0.93-3.97) vs 1.08 (0.75-2.38)	p=0.934 0.939 (0.471-1.447) vs 1.002 (0.254-2.608)	p=0.782 1.871 (0.784-3.676) vs 2.169 (0.751-4.112)	p=0.912 2.02 (0.94-3.97) vs 2.24 (0.75-4.11)	p=0.959 2.14 (0.78-3.68) vs 2.24 (0.93-4.11)	p=0.662 2.21 (0.93-4.11) vs 2.10 (0.75-3.97)	p=0.677 2.14 (0.93-4.11) vs 2.29 (0.75-3.96)	p=0.163 1.61 (0.94-2.79) vs 2.74 (0.75-4.11)
**CAT**	p=0.467 0.044 (0.013-0.097) vs 0.065 (0.015-0.1500)	p=0.635 0.201 (0.056-0.359) vs 0.152 (0.031-0.421)	p=0.795 0.06 (0.01-0.15) vs 0.05 (0.04-0.13)	***p<0.05*** 0.16 (0.06-0.41) vs 0.13 (0.03-0.16)	p=0.094 0.04 (0.01-0.09) vs 0.07 (0.02-0.15)	p=0.371 0.17 (0.06-0.36) vs 0.14 (0.03-0.42)	p=0.669 0.05 (0.01-0.13) vs 0.07 (0.02-0.15)	p=0.158 0.13 (0.05-0.31) vs 0.18 (0.13-0.42)	p=0.591 0.06 (0.02-0.13) vs 0.06 (0.02-0.07)	p=0.391 0.16 (0.05-0.36) vs 0.13 (0.13-0.15)	p=0.934 0.056 (0.012-0.132) vs 0.068 (0.018-0.15)	p=0.858 0.135 (0.052-0.359) vs 0.141 (0.120-0.228)	p=0.492 0.17 (0.03-0.36) vs 0.14 (0.05-0.42)	p=0.898 0.15 (0.05-0.32) vs 0.14 (0.06-0.42)	***p<0.05*** 0.13 (0.03-0.36) vs 0.18 (0.09-0.42)	***p<0.05*** 0.13 (0.03-0.35) vs 0.18 (0.05-0.42)	p=0.223 0.16 (0.13-0.32) vs 0.13 (0.03-0.42)

**GPx**	p=0.155 0.183 (0.169-0.189) vs 0.223 (0.137-0.399)	***p=0.029*** 0.166 (0.147-0.194) vs 0.232 (0.127-0.304)	p=0.260 0.23 (0.14-0.39) vs 0.19 (0.16-0.21)	p=0.236 0.23 (0.13-0.30) vs 0.19 (0.15-0.25)	p=0.836 0.21 (0.16-0.33) vs 0.21 (0.14-0.39)	p=0.982 0.21 (0.15-0.30) vs 0.21 (0.13-0.30)	p=0.357 0.22 (0.14-0.39) vs 0.21 (0.16-0.28)	p=0.544 0.23 (0.15-0.30) vs 0.19 (0.13-0.30)	p=0.802 0.21 (0.16-0.33) vs 0.22 (0.16-0.38)	p=0.971 0.21 (0.15-0.30) vs 0.23 (0.19-0.24)	p=0.114 0.232 (0.169-0.399) vs 0.185 (0.157-0.383)	p=0.760 0.232 (0.147-0.304) vs 0.197 (0.127-0.271)	p=0.438 0.23 (0.15-0.30) vs 0.19 (0.13-0.27)	p=0.212 0.23 (0.15-0.30) vs 0.19 (0.13-0.25)	p=0.348 0.23 (0.13-0.30) vs 0.19 (0.15-0.30)	p=0.417 0.23 (0.13-0.30) vs 0.19 (0.15-0.25)	p=0.080 0.24 (0.15-0.30) vs 0.19 (0.13-0.25)
**GR**	p=0.635 23.31 (19.89-27.06) vs 25.41 (18.87-39.97)	p=0.327 23.91 (20.76-27.05) vs 26.74 (19.14-42.75)	p=0.312 25.68 (19.01-39.21) vs 23.50 (19.89-25.97)	p=0.078 26.77 (19.74-41.15) vs 24.21 (20.76-28.16)	p=0.697 25.22 (19.89-33.40) vs 25.41 (18.87-39.97)	p=0.945 26.76 (20.76-36.34) vs 26.73 (19.14-42.75)	p=0.271 25.70 (19.60-39.97) vs 24.30 (18.87-29.68)	p=0.252 26.80 (20.76-36.34) vs 26.70 (19.14-42.75)	p=0.415 25.18 (19.42-33.40) vs 25.414 (24.36-39.97)	p=0.410 26.77 (20.76-36.34) vs 26.70 (24.21-26.72)	p=0.389 25.71 (19.60-36.90) vs 24.33 (18.87-39.97)	p=0.081 26.81 (20.76-36.34) vs 26.54 (19.14-26.83)	p=0.177 26.91 (21.04-42.75) vs 26.72 (19.14-29.74)	p=0.078 26.78 (21.54-36.34) vs 24.21 (19.14-29.74)	p=0.285 26.77 (19.14-36.34) vs 26.72 (20.76-42.74)	p=0.614 26.75 (19.14-42.75) vs 26,74 (20.76-29.38)	p=0.579 26.76 (21.04-42.75) vs 26.74 (19.14-29.74)
**GSH**	***p=0.015*** 0.668 (0.097-5.493 vs 1.373 (0.558-0.955)	p=0.884 1.491 (1.170-2.267) vs 1.348 (0.332-4.184)	p=0.112 1.33 (0.21-4.97) vs 0.82 (0.53-1.49)	p=0.839 1.61 (0.40-3.88) vs 1.35 (1.13-3.06)	p=0.872 1.43 (0.09-2.46) vs 1.07 (0.56-5.49)	p=0.582 1.26 (0.78-4.18) vs 1.91 (0.33-3.06)	p=0.840 1.26 (0.09-5.49) vs 1.07 (0.56-2.30)	p=0.893 1.91 (0.61-2.98) vs 1.33 (0.33-4.18)	p=0.267 1.07 (0.53-3.41) vs 1.49 (1.06-2.96)	p=0.802 1.48 (0.76-3.06) vs 1.35 (0.61-2.27)	p=0.718 1.156 (0.096-5.493) vs 1.353 (0.558-2.961)	p=0.803 1.310 (0.332-4.184) vs 1.758 (0.606-2.980)	p=0.507 1.24 (0.78-4.18) vs 1.91 (0.33-3.06)	p=0.979 1.34 (0.61-3.06) vs 1.60 (0.79-2.19)	p=0.930 1.46 (0.61-4.18) vs 1.36 (0.33-3.06)	p=0.599 1.54 (0.33-4.26) vs 1.36 (0.77-3.06)	p=0.611 1.25 (0.61-4.18) vs 1.62 (0.33-2.98)
**GSSG**	p=0.681 0.163 (0.093-0.317) vs 0.144 (0.009-0.424)	p=0.327 0.229 (0.170-0.349) vs 0.301 (0.115-0.456)	p=0.874 0.144 (0.009-0.424) vs 0.157 (0.093-0.246)	p=0.635 0.300 (0.127-0.456) vs 0.259 (0.115-0.374)	p=0.396 0.174 (0.077-0.316) vs 0.135 (0.009-0.424)	p=0.477 0.293 (0.115-0.367) vs 0.300 (0.127-0.456)	p=0.291 0.168 (0.118-0.424) vs 0.131 (0.009-0.301)	p=0.312 0.306 (0.115-0.456) vs 0.276 (0.127-0.419)	p=0.971 0.139 (0.009-0.424) vs 0.178 (0.013-0.246)	p=0.163 0.301 (0.127-0.456) vs 0.155 (0.115-0.339)	p=0.085 0.182 (0.035-0.424) vs 0.122 (0.009-0.301)	p=0.058 0.309 (0.170-0.456) vs 0.259 (0.115-0.419)	p=0.674 0.306 (0.127-0.419) vs 0.295 (0.115-0.456)	p=0.832 0.295 (0.127-0.456) vs 0.300 (0.115-0.426)	p=0.983 0.301 (0.134-0.426) vs 0.294 (0.115-0.456)	p=0.368 0.289 (0.127-0.426) vs 0.339 (0.115-0.456)	p=0.177 0.293 (0.115-0.410) vs 0.300 (0.167-0.456)
**Redox status**	***p=0.008*** 2.947 (0.048-4.913) vs 14.77 (1.511-659.7)	p=0.728 11.23 (3.912-22.27) vs 10.08 (0.374-61.67)	p=0.106 14.77 (0.048-659.7) vs 4.311 (3.018-8.946)	***p=0.017*** 0.300 (0.127-0.456) vs 13.35 (3.912-24.96)	p=0.477 10.61 (0.048-53.39) vs 14.58 (1.511-659.7)	p=0.908 7.424 (1.633-61.67) vs 11.22 (0.374-32.15)	***p<0.0001*** 16.20 (0.048-659.7) vs 0.276 (0.127-0.418)	p=0.637 10.50 (0.374-61.67) vs 12.97 (2.379-32.15)	p=0.591 13.43 (0.048-214.1) vs 8.946 (6.381-159.7)	p=0.915 10.50 (0.374-61.67) vs 15.15 (2.379-15.79)	p=0.678 12.28 (0.048-71.14) vs 8.462 (2.545-56.00)	p=0.326 4.736 (0.374-61.67) vs 13.00 (2.379-32.15)	p=0.521 7.406 (1.633-61.67) vs 12.97 (0.374-32.15)	p=0.916 10.92 (0.374-61.67) vs 11.22 (2.733-15.79)	p=0.913 9.316 (2.379-61.67) vs 11.54 (0.374-31.35)	p=0.982 10.65 (0.37-61.67) vs 10.92 (1.63-24.96)	p=0.842 10.50 (2.323-61.67) vs 11.22 (0.374-32.15)
**TAC**	***p=0.046*** 1.103 (0.982-1.157) vs 1.283 (0.898-2.024)	p=0.635 1.316 (1.081-1.712) vs 1.427 (1.049-2.293)	p=0.403 1.25 (0.92-1.98) vs 1.18 (1.09-1.29)	p=0.126 1.44 (1.05-2.18) vs 1.34 (1.08-1.44)	p=0.566 1.16 (0.98-1.49) vs 1.22 (0.89-2.02)	p=0.836 1.41 (1.08-1.74) vs 1.44 (1.05-2.29)	p=0.357 1.24 (0.98-2.02) vs 1.19 (0.89-1.43)	p=0.805 1.43 (1.07-1.83) vs 1.42 (1.05-2.29)	p=0.123 1.19 (0.97-1.69) vs 1.32 (1.29-2.02)	p=0.185 1.44 (1.06-1.83) vs 1.34 (1.19-1.36)	p=0.889 1.183 (0.982-1.851) vs 1.255 (0.898-2.024)	p=0.360 1.430 (1.065-1.737) vs 1.349 (1.049-1.590)	p=0.982 1.43 (1.07-2.29) vs 1.44 (1.05-1.71)	p=0.345 1.43 (1.08-1.83) vs 1.36 (1.05-1.69)	p=0.230 1.45 (1.05-1.83) vs 1.36 (1.06-2.29)	p=0.303 1.44 (1.05-2.29) vs 1.37 (1.08-1.71)	p=0.912 1.41 (1.05-2.29) vs 1.43 (1.17-1.71)
**TOS**	p=0.071 1.195 (1.091-5.035) vs 4.543 (0.601-11.87)	p=0.924 5.228 (4.311-6.198) vs 5.123 (0.401-12.50)	***p<0.001*** 4.39 (0.62-9.88) vs 30.40 (8.17-85.25)	p=0.885 5.16 (0.61-11.74) vs 4.31 (3.66-11.14)	p=0.630 4.48 (0.60-10.46) vs 4.39 (0.67-11.87)	p=0.422 4.712 (2.13-11.14) vs 5.20 (0.40-12.50)	p=0.770 4.39 (0.60-11.87) vs 4.54 (0.67-7.81)	p=0.702 5.02 (1.24-12.50) vs 6.14 (0.40-8.92)	p=0.857 4.39 (0.67-10.46) vs 4.57 (2.22-7.71)	p=0.333 5.10 (1.24-9.48) vs 7.73 (3.34-11.14)	p=0.637 4.195 (0.601-11.87) vs 4.629 (1.746-7.813)	p=0.063 4.739 (1.238-12.50) vs 6.394 (3.334-11.14)	p=0.116 4.71 (0.40-12.50) vs 5.72 (3.01-11.14)	p=0.070 4.83 (1.24-9.48) vs 6.39 (4.31-11.14)	p=0.844 5.06 (0.40-12.50) vs 5.12 (1.24-11.14)	p=0.417 4.99 (0.41-9.48) vs 5.72 (3.01-12.56)	p=0.176 4.61 (1.24-11.14) vs 5.72 (0.40-12.50)
**OSI**	p=0.082 1.055 (0.991-5.127) vs 3.257 (0.526-10.03)	p=0.824 3.463 (3.315-5.736) 3.740 (1.162-8.640)	p=0.977 3.09 (0.51-8.48) vs 3.55 (0.99-10.03)	p=0.583 3.35 (0.50-8.10) vs 3.59 (2.54-8.21)	p=0.663 3.24 (0.53-9.04) vs 3.24 (0.50-10.03)	p=0.909 3.35 (1.23-8.21) vs 3.74 (0.28-8.64)	p=0.911 3.09 (0.53-10.03) vs 3.29 (1.69-6.54)	p=0.515 3.35 (1.16-8.64) vs 4.43 (2.48-6.14)	p=0.971 3.09 (0.99-9.04) vs 3.55 (1.69-3.81)	p=0.299 3.48 (1.23-6.14) vs 6.49 (2.48-8.21)	p=0.560 2.566 (0.526-10.03) vs 3.418 (1.686-6.538)	p=0.056 3.339 (1.162-8.640) vs 5.042 (2.484-8.212)	p=0.223 3.21 (1.16-8.64) vs 4.04 (2.07-8.21)	***p<0.05*** 3.33 (1.23-6.49) vs 5.23 (3.08-8.21)	p=0.948 3.89 (1.23-8.64) vs 3.59 (1.16-8.21)	p=0.245 3.24 (1.16-6.13) vs 3.74 (2.07-8.75)	***p<0.05*** 2.77 (1.16-8.21) vs 4.43 (2.07-8.63)
**AGE**	p=0.054 0.481 (0.336-0.647) vs 0.715 (0.422-1.174)	p=0.242 0.685 (0.541-1.120) vs 0.891 (0.106-2.083)	p=0.839 0.65 (0.36-1.14) vs 0.65 (0.44-0.99)	p=0.112 0.94 (0.20-1.93) vs 0.61 (0.54-1.17)	p=0.069 0.57 (0.34-0.93) vs 0.77 (0.42-1.17)	p=0.449 0.79 (0.11-1.47) vs 0.89 (0.49-2.08)	p=0.200 0.61 (0.34-1.02) vs 0.84 (0.42-1.17)	p=0.170 1.02 (0.54-2.08) vs 0.78 (0.11-1.25)	p=0.915 0.65 (0.37-1.12) vs 0.81 (0.47-0.84)	p=0.410 0.84 (0.24-1.87) vs 1.25 (0.61-1.43)	p=0.802 0.605 (0.336-1.018) vs 0.645 (0.422-1.174)	p=0.233 1.070 (0.541-2.083) vs 0.794 (0.107-1.431)	p=0.465 0.94 (0.55-1.47) vs 0.82 (0.11-2.08)	***p<0.05*** 0.61 (0.49-0.98) vs 1.02 (0.55-1.47)	p=0.879 0.87 (0.11-1.47) vs 0.82 (0.54-2.08)	p=0.742 0.83 (0.07-1.41) vs 0.98 (0.51-2.17)	p=0.739 0.832 (0.107-1.470) vs 0.851 (0.494-2.083)

**AOPP**	p=0.429 6.276 (1.676-10.16) vs 7.787 (1.445-18.35)	***p=0.027*** 10.49 (9.095-11.20) vs 13.32 (7.395-26.37)	p=0.977 7.31 (1.47-17.83) vs 7.93 (3.86-10.16)	p=0.285 13.04 (7.47-25.02) vs 10.36 (9.09-22.35)	p=0.176 6.09 (1.67-16.27) vs 7.93 (1.45-18.36)	***p<0.05*** 11.02 (7.39-26.37) vs 13.61 (7.68-22.36)	p=0.357 6.28 (1.68-16.27) vs 11.71 (1.45-18.35)	p=0.964 12.50 (8.47-26.37) vs 12.16 (7.39-20.96)	p=0.857 7.31 (1.55-16.27) vs 8.52 (2.52-12.20)	p=0.693 12.20 (7.67-22.35) vs 14.34 (10.36-15.52)	p=0.846 6.126 (1.676-16.27) vs 7.737 (1.445-18.35)	p=0.739 12.77 (8.469-26.37) vs 12.55 (7.395-20.95)	p=0.982 12.20 (10.16-26.37) vs 13.61 (7.39-22.35)	p=0.221 12.50 (8.48-22.34) vs 11.20 (7.68-14.32)	p=0.727 12.77 (7.39-26.37) vs 12.02 (8.47-22.35)	p=0.154 13.46 (7.39-26.37) vs 11.20 (7.47-22.45)	p=0.808 12.36 (7.39-26.37) vs 12.24 (8.47-20.95)
**MDA**	p=0.062 0.501 (0.421-0.608) vs 0.660 (0.210-2.184)	p=0.728 0.909 (0.641-1.458) vs 0.845 (0.478-1.718)	p=0.665 0.65 (0.25-1.97) vs 0.57 (0.48-0.96)	p=0.839 0.87 (0.48-1.66) vs 0.84 (0.64-1.05)	p=0.231 0.55 (0.21-0.72) vs 0.69 (0.38-2.18)	p=0.836 0.89 (0.59-1.46) vs 0.85 (0.48-1.72)	p=0.669 0.61 (0.21-2.18) vs 0.66 (0.38-1.21)	**p<0.05** 0.58 (0.27-0.93) vs 0.93 (0.85-1.07)	p=0.858 0.63 (0.38-1.34) vs 0.59 (0.48-0.83)	p=0.911 0.87 (0.48 1.50) vs 0.85 (0.49-1.72)	p=0.846 0.608 (0.210-2.184) vs 0.600 (0.377-0.828)	p=0.360 0.891 (0.514-1.458) vs 0.783 (0.477-1.265)	p=0.465 0.91 (0.51-1.72) vs 0.81 (0.48-1.46)	p=0.979 0.87 (0.51-1.50) vs 0.81 (0.49-1.42)	p=0.445 1.02 (0.49-1.50) vs 0.84 (0.48-1.72)	p=0.263 0.92 (0.49-1.72) vs 0.84 (0.48-1.46)	**p<0.001** 0.61 (0.21-1.21) vs 0.85 (0.48-1.46)
**8-OHDG**	p=0.359 0.772 (0.736-0.823) vs 0.867 (0.516-1.438)	p=0.071 0.920 9 (0.747-1.023) vs 1.119 (0.704-1.684)	p=0.931 0.80 (0.54-1.40) vs 0.82 (0.73-0.93)	***p<0.05*** 1.12 (0.73-1.62) vs 0.96 (0.75-1.11)	p=0.766 0.81 (0.73-0.97) vs 0.87 (0.52-1.44)	p=0.136 1.022 (0.75-1.15) vs 1.15 (0.70-1.68)	p=0.271 0.85 (0.65-1.44) vs 0.78 (0.52-0.97)	p=0.458 1.04 (0.75-1.40) vs 1.15 (0.70-1.68)	p=0.371 0.81 (0.63-1.11) vs 0.97 (0.73-1.29)	p=0.543 1.07 (0.75-1.40) vs 1.12 (0.97-1.45)	p=0.305 0.867 (0.654-1.438) vs 0.776 (0.630-1.285)	p=0.718 1.023 (0.747-1.400) vs 1.101 (0.704-1.446)	p=0.912 1.04 (0.80-1.68) vs 1.11 (0.70-1.45)	p=0.429 1.09 (0.82-1.45) vs 0.97 (0.70-1.40)	p=0.169 1.14 (0.70-1.45) vs 1.02 (0.75-1.68)	p=0.181 1.14 (0.70-1.68) vs 1.02 (0.75-1.22)	p=0.808 1.07 (0.70-1.68) vs 1.12 (0.75-1.40)

Table S2. Comparison of oxidative stress biomarkers between tumour tissue and normal adjacent mucosa.

Parameter	Tumour vs non-tumour tissue		p-value
**NOX**	6.128 (4.941-12.06)	4.774 (1.660-11.26)	**0.0012**
**XO**	13.53 (10.17-21.05)	12.01 (9.466-18.84)	**0.0146**
**SOD**	2.178 (0.751-4.112)	0.986 (0.254-2.608)	**< 0.0001**
**CAT**	0.152 (0.031-0.422)	0.057 (0.013-0.150)	**< 0.0001**
**GPx**	0.214 (0.126-0.304)	0.205 (0.137-0.399)	0.901
**GR**	26.74 (19.14-42.75)	25.41 (18.87-39.97)	0.192
**GSH**	1.362 (0.332-4.184)	1.156 (0.097-3.493)	0.214
**TAC**	1.427 (1.049-2.293)	1.199 (0.898-2.024)	**0.009**
**TOS**	5.123 (0.601-12.87)	4.394 (0.401-11.51)	0.198
**OSI**	3.362 (0.281-8.640)	3.239 (0.101-7.457)	0.327
**AGE**	0.871 (0.106-2.083)	0.646 (0.336-1.174)	**0.0034**
**AOPP**	12.24 (7.395-26.37)	7.675 (1.445-18.35)	**0.0004**
**MDA**	0.853 (0.477-1.718)	0.615 (0.210-1.284)	**0.0007**
**8-OHDG**	1.083 (0.704-1.684)	0.823 (0.516-1.438)	**< 0.0001**

© 2020 by the authors. Licensee MDPI, Basel, Switzerland. This article is an open access article distributed under the terms and conditions of the Creative Commons Attribution (CC BY) license (http://creativecommons.org/licenses/by/4.0/).
